# The Combined Use of *Schizosaccharomyces pombe* and *Lachancea thermotolerans*—Effect on the Anthocyanin Wine Composition

**DOI:** 10.3390/molecules22050739

**Published:** 2017-05-04

**Authors:** Ángel Benito, Fernando Calderón, Santiago Benito

**Affiliations:** Departamento de Química y Tecnología de Alimentos, Universidad Politécnica de Madrid, Ciudad Universitaria S/N, 28040 Madrid, Spain; angel@urbinavinos.com (Á.B.); fernando.calderon@upm.es (F.C.)

**Keywords:** fission yeast, *Schizosaccharomyces pombe*, *Lachancea thermotolerans*, pyruvic acid, malic acid, lactic acid, urea, ethyl carbamate, biogenic amines, anthocyanins, winemaking

## Abstract

The most popular methodology to make red wine is through the combined use of *Saccharomyces cerevisiae* yeast and lactic acid bacteria, for alcoholic fermentation and malolactic fermentation respectively. This classic winemaking practice produces stable red wines from a microbiological point of view. This study aims to investigate a recent red winemaking biotechnology, which through the combined use of *Lachancea thermotolerans* and *Schizosaccharomyces pombe* is used as an alternative to the classic malolactic fermentation. In this new methodology, *Schizosaccharomyces* *pombe* totally consumes malic acid, while *Lachancea thermotolerans* produces lactic acid, avoiding excessive deacidification of musts with low acidity in warm viticulture areas such as Spain. This new methodology has been reported to be a positive alternative to malolactic fermentation in low acidity wines, since it has the advantage to produce wines with a more fruity flavor, less acetic acid, less ethyl carbamate originators and less biogenic amines than the traditional wines produced via conventional fermentation techniques. The study focuses on unexplored facts related to this novel biotechnology such as color and anthocyanin profile.

## 1. Introduction

Until recently, classic alcoholic fermentation and malolactic fermentation were considered to be the unique methodologies to obtain stable red wine from a microbiological point of view before bottling [1,2,3,4]. Several researchers are now paying special attention to the use of non-*Saccharomyces* yeasts in oenology to improve wine quality [5,6,7,8]. These new biotechnologies are generating new trends in wine microbiology, to improve wine quality. Some of the most studied non-*Saccharomyces* yeast species in winemaking, are *Candida zemplinina* [9], *Torulaspora delbrueckii* [10,11], *Kloeckera apiculata* [12], *Hanseniaspora vineae* [13], *Hanseniaspora uvarum* [14], *Candida pulcherrima* [15], *Hansenula anomala* [16], *Schizosaccharomyces pombe* (*S. pombe*) [3,4], and *Lachancea thermotolerans* (*L. thermotolerans*) [17,18]. Most of these studies, report sequential inoculation’s of a non-*Saccharomyces* and a *Saccharomyces cerevisiae* to produce the best improvements in wine quality.

*S. pombe* was traditionally used for deacidification due to the fact that it can convert harsh tasting l-malic acid into ethanol, making very acidic wines smoother [19,20,21]. Nevertheless, microorganisms of the genus *Schizosaccharomyces* are being used nowadays to reach other objectives in modern winemaking. One new use, involves procedures that cause a high polysaccharide release during fermentation [4,22] and ageing over lees [22]. Another use, is decreasing the gluconic acid levels from initial grape juice [23] in order to increase wine quality in spoiled musts. *S. pombe* alone has also been verified to improve the color of red wines, because it increases the content of highly stable pigments such as vitisins and pyranoanthocyanin [24,25,26]. Finally, from a food safety viewpoint, the genus *Schizosaccharomyces* is being used to produce safer wines, because it possesses urease activity [27] that avoids ethyl carbamate production and reduces the risk of biogenic amine formation by wild lactic acid bacteria [1]. Conversely, *Lachancea thermotolerans* (*L. thermotolerans*) is used to produce more acidic wines in warm regions from low acidic musts [18,28,29,30].

The species *S. pombe* has not been traditionally used for winemaking [31,32,33] due to the existence of some collateral effects caused by metabolites such as acetic acid, acetaldehyde, acetoin and ethyl acetate [34]. Those problems have been solved recently through the performance of improved strain selection processes [3,35]. The main issue regarding the selection processes was the difficulty in isolating a representative number of strains from environmental samples [36], thus limiting the ability to obtain and collect representative strains of *Schizosaccharomyces* genus [37]. The number of strains available is currently very low compared to *Saccharomyces cerevisiae* (*S. cerevisiae*) strains offer; Thus further selection processes similar to those performed for *S. cerevisiae* in winemaking would be required in the future [38,39,40].

New biotechnology involving the combined use of *L. thermotolerans* and *S. pombe* has been studied before regarding basic winemaking parameters [1] and advanced factors such as aroma volatiles, amino acids or food safety factors [2]. Nevertheless, many other unexplored wine parameters need to be studied for this novel biotechnology. This specific study is focused on the influence of the combined use of *L. thermotolerans* and *S. pombe* on wine anthocyanin composition.

## 2. Results and Discussion

### 2.1. Fermentation Kinetics

#### 2.1.1. Yeast Population Kinetics

**Figure 1:**
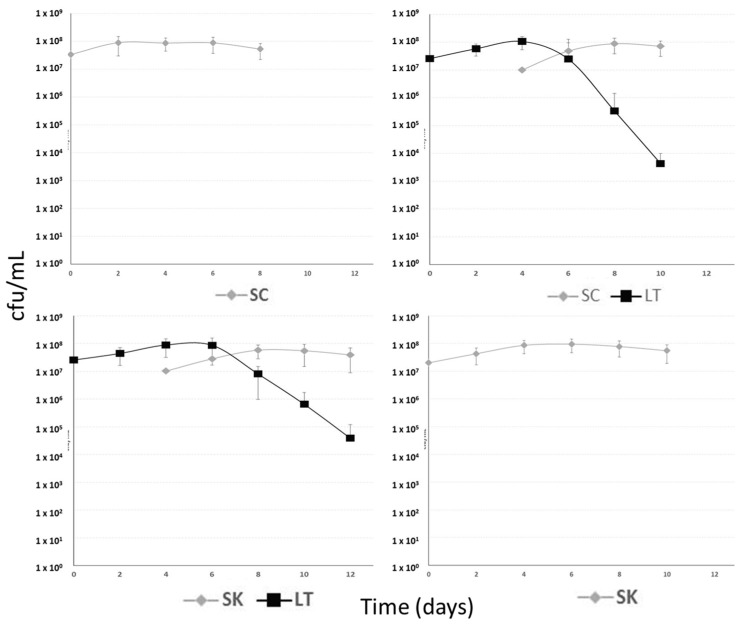
Population development during fermentation of *S. cerevisiae* 88 alone (SC), sequential fermentation with *Saccharomyces cerevisiae* 88 and *L. thermotolerans* CONCERTO™ (LT…SC), sequential fermentation with *S. pombe* V2 and *Lachancea thermotolerans* CONCERTO™ (LT…SK) and *S. pombe* V2 alone (SK).

Figure 1 shows the growth of the different yeast strains during fermentation. In sequential fermentations, inoculated with *Saccharomyces cerevisiae* 88 or *S. pombe* V2, *L. thermotolerans* CONCERTO™ started to decline just after the second inoculation, although the *L. thermotolerans* population decrease was more rapid in the presence of *S. cerevisiae*. The progressive disappearance of *L. thermotolerans* could be explained as a result of the presence of another more well-adapted yeast competitor (*S. cerevisiae* or *S. pombe*) and an ethanol concentration of about 9% *v*/*v* by day 6. *L. thermotolerans* has been reported to tolerate 9% *v*/*v* ethanol in a pure culture fermentation [28]. This limited alcohol tolerance of *L. thermotolerans* causes difficulties in the production of a dry red wine in warm regions only without using other yeast with higher ethanol tolerance in a combined fermentation.

#### 2.1.2. Sugar Consumption Kinetics

**Figure 2:**
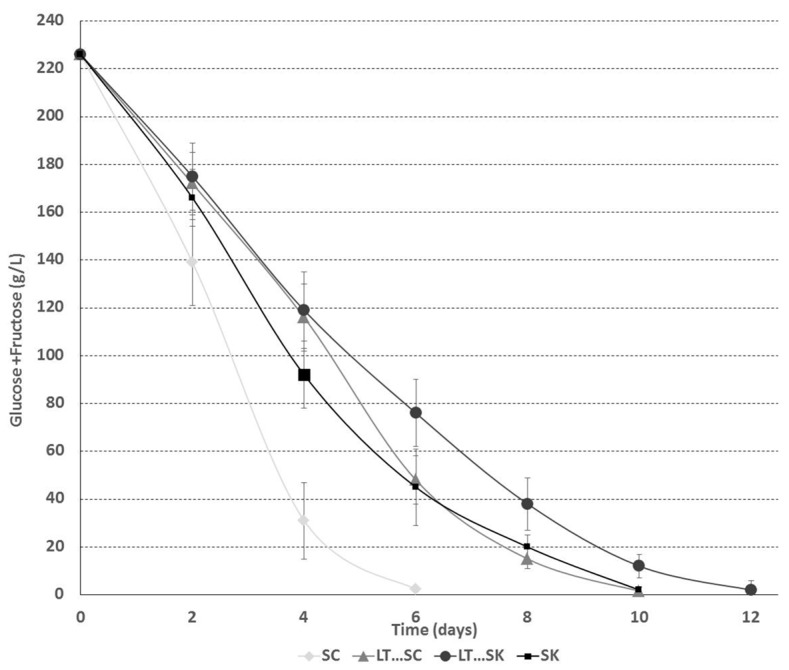
Fermentation kinetics of glucose + fructose for *Saccharomyces cerevisiae* 88 alone (SC), a sequential fermentation with *Saccharomyces cerevisiae* 88 and *L. thermotolerans* CONCERTO™ (LT…SC), a sequential fermentation with *Schizosaccharomyces pombe* V2 and *L. thermotolerans* CONCERTO™ (LT…SK) and *Schizosaccharomyces pombe* V2 alone (SK).

The consumption kinetics of glucose and fructose were more rapid when *S. cerevisiae* strain 88 was involved (Figure 2) than when *L. thermotolerans* and *S. pombe* were used. The alcoholic fermentation times varied from 6 to 12 days. All alcoholic fermentations finished correctly, reaching concentrations lower than 2 g/L of glucose and fructose (Figure 2; Table 1). Other authors have previously described slower fermentation kinetics for *L. thermotolerans* [17,18] and *S. pombe* [31] than for *S. cerevisiae*. Musts with high sugar contents have been reported to be improperly fermented by *L. thermotolerans* alone [28].

### 2.2. Acetic Acid

The maximal final concentration of acetic acid was 0.43 g/L for a malolactic fermentation following an alcoholic fermentation by *S. cerevisiae* in pure culture (Table 1). Alcoholic fermentations alone did not show significant differences, with values about 0.31 g/L. Previous studies reported that *L. thermotolerans* produced less acetic acid than *S. cerevisiae*, with those differences varing from 0.18 to 0.33 g/L [30,41]. The genus *Schizosaccharomyces* was previously reported as producing more acetic acid than *S. cerevisiae*, with acetic acid concentrations up to 1 g/L [25]. Nevertheless, some *S. pombe* strains have been recently selected for their low acetic acid production [3,35], and the results for those strains agree with the results obtained in this study.

### 2.3. Malic Acid

Malic acid was completely degraded in all trials involving *S. pombe* (Table 1) during alcoholic fermentation. The *S. cerevisiae* strain degraded 7% of the initial malic acid content in the must (Table 1). Several authors have reported similar malic acid degradation for yeast of other genus than *Schizosaccharomyces*, which varied up to 20% [10,35] or even up to 39% for specific hybrids [42], but no one has reported the total degradation of malic acid (i.e., 100%) unless *Schizosaccharomyces* genus is involved [21]. The malic acid reduction clearly affected the final pH value of the fermentations (Table 1) because *S. pombe* fermentations reached a final pH up to 3.88. *O. oeni* metabolized malic acid to lactic acid during malolactic fermentation (Table 1).

### 2.4. l-Lactic Acid

Fermentations involving *L. thermotolerans* produced l-lactic acid during alcoholic fermentation (Table 1). The final concentration of l-lactic acid produced by *L. thermotolerans* in this study varied from 2.44 to 2.77 g/L (Table 1), which reduced the final pH (Table 1). Previous studies have reported significant acidification from l-lactic acid, varying from 0.22 g/L to 6.38 g/L when mixed cultures of *L. thermotolerans* were used with the main objective of increasing the acidity of the must [18,30,43]. Experiments involving malolactic fermentations showed an increase in l-lactic acid of approximately 1.21 g/L (Table 1). These final l-lactic acid concentration levels were significantly lower than the ones obtained using *L. thermotolerans* for the studied must.

### 2.5. Pyruvic Acid

All fermentations involving *S. pombe* produced a higher pyruvic acid concentration than the others (Table 1). The maximum values are usually obtained during the first days of alcoholic fermentation [3,4]. The pure culture of *S. pombe* produced, after alcoholic fermentation, a final pyruvic acid concentration of 286 mg/L. Greater pyruvic acid formation was associated to the higher color intensity observed in this study for *S. pombe* fermentations, due to the involvement of this compound in the formation of highly stable colored pigments such as vitisin A [18,25,26,44]. 

### 2.6. Glycerol

The genera *Schizosaccharomyces* and *L.* have been described as higher glycerol producers than the genus *Saccharomyces* [18,30,41]. The final levels of glycerol varied from 6.43 g/L to 7.55 g/L (Table 1). *S. pombe* produced the highest concentration (Table 1). A high glycerol content has been described as one of the main contributions of non-*Saccharomyces* strains to wine quality [5,45,46]. Nevertheless, other authors have reported that species such as *Candida stellata* could effectively produce higher concentrations of glycerol up to 14 g/L [5].

### 2.7. Ethanol

The ethanol levels varied from 12.83 to 13.27% (*v/v*) (Table 1). Other authors have reported that *S. pombe* is highly resistant to ethanol stress conditions [47]. Sugar metabolism can be used to synthetize compounds other than ethanol, such as glycerol or pyruvic acid, or to increase the biomass of the yeast [48,49]. The results show that fermentations involving *L. thermotolerans* and *S. pombe* produced lower ethanol levels than *S. cerevisiae*. These data accords to other authors who confirmed that some non-*Saccharomyces* yeasts produced lower ethanol yields than *Saccharomyces* [23,50,51,52,53]. Previous studies have shown similar results for *L. thermotolerans* [30] and *S. pombe* [50]. Nevertheless, the differences (Table 1) were approximately 0.44% (*v/v*). Some authors have recently reported more significant ethanol reductions greater than 1% (*v/v*) using non-*Saccharomyces* strains, which may be related to specific conditions of high aeration [54,55] or via the use of glucose oxidase and catalase [56].

### 2.8. Urea

The final concentration of urea in the completed alcoholic fermentations was lower in fermentations involving *S. pombe*, with values less than 0.1 mg/L (Table 1). This effect was attributed to the enzymatic capacity of *Schizosaccharomyces* to produce urease [27,57], whose enzymatic activity was proposed in the past as a preventative measure to the hazard of carcinogenic ethyl carbamate formation (one of the most toxic compounds reported in wine) [3,58] in winemaking because urease eliminates urea, the main precursor of ethyl carbamate. This factor is becoming increasingly important because ethyl carbamate is a known carcinogen present in a variety of fermented foods [59]. Some countries such as the USA, Japan and Canada have established legal limits.

### 2.9. Citric Acid

No statistical differences in citric acid were observed during any alcoholic fermentation (Table 1). However, in the experiment in which *O. oeni* was inoculated after an alcoholic fermentation, most of the citric acid was consumed (Table 1). An increase in the acetic acid concentration was also detected during the same period. Citric acid is converted to diacetyl, acetoin and 2,3-butanediol. Acetic acid is a by-product in that process; such a collateral effect usually increases the final acetic acid concentration [25,60].

### 2.10. Color Measurements

Table 2 shows the results of color assessments for the different treatments. Fermentations regarding *S. pombe* alone showed the highest color intensity levels up to 0.22. Combined fermentation between *S. pombe* and *L. thermotolerans* showed the second higher color intensity up to 0.20. Color intensity decreased up to 22% and hue increased in 0.34 in fermentations where malolactic fermentation took place (Table 2).

### 2.11. Anthocyanins

Table 3 shows the values for anthocyanins in the different fermentations. Fermentations involving *S. pombe* alone showed the highest concentration values in vitisin A up to 5.98 mg/L. A significant decrease of about 1.33 mg/L in vitisin A was detected for the wines that performed malolactic fermentation. The highest vitisin A concentration values were reported in those fermentations that showed the highest pyruvic acid concentrations (Table 1). The lowest concentration in Vitisin A was reported for the *S. cerevisiae* assay which performed malolactic fermentation, probably, due to the decrease of pyruvic acid concentration reported during this process (Table 1). Vitisin B decreased about 0.38 mg/L for the treatments which underwent malolactic fermentation, which is explained by the high decrease in acetaldehyde which took place during that process (Table 1). Vitisin B levels are related to the acetaldehyde concentrations observed for the different trials (Table 1). The *S. pombe* assays showed significant higher values than the *S. cerevisiae* treatments. The fermentation performed by *S. cerevisiae* and *L. thermotolerans* showed lower acetaldehyde concentration, with a similar phenomenon being reported in the past [43]. The formation of highly stable pigments such as vitisins is connected with improved chromatic characteristics of the wine, especially during long ageing process when stable pigment forms start to dominate over unstable forms. On the other hand malvidin-3-*O*-glucoside dropped significantly up to 30 mg/L after the malolactic fermentation process. The assays that did not perform malolactic fermentation showed significantly higher values in all the studied anthocyanins. Those wines also showed higher color intensity values (Table 2). Indeed, important decreases in the total sum of anthocyanins were detected after malolactic fermentation for trials that did not involve the use of *S. pombe* (Table 3). Those drops varied from 26 to 31%. High anthocyanin levels are connected with better color quality, improved mouthfeel and a better ageing potential. Combined fermentation between *S. pombe* and *L. thermotolerans* showed higher concentrations in grape anthocyanins such as delphinidin-3-*O*-glucoside, cyanidin-3-*O*-glucoside, petunidin-3-*O*-glucoside, peonidin-3-*O*-glucoside or malvidin-3-*O*-glucoside, than when *S. pombe* fermented alone. Combined fermentation involving *S. cerevisiae* and *L. thermotolerans* also showed higher levels of grape anthocyanins than when *S. cerevisiae* fermented alone. This phenomenon could be related to a low anthocyanin absorption by *L. thermotolerans* strain. The higher levels in coumaroyl compounds such as cyanidin-3-*O*-(6′′-*p*-coumaroylglucoside), petunidin-3-*O*-(6′′-*p*-coumaroylglucoside) or malvidin-3-*O*-(6′′-*p*-coumaroylglucoside), reported for combined fermentations (Table 3) when compared to fermentations performed by *S. cerevisiae* and *S. pombe* alone, can be also explained by lower absorption processes. No vinyl phenol pyranoanthocyanins were detected for any treatment, probably becauset that studied strains do not possess any hydroxycinnamate decarboxylase activity [46,61]. The low concentrations detected for some anthocyanins could be related to the effect of heat treatment of the initial must [62].

### 2.12. Sensory Evaluation

**Figure 3:**
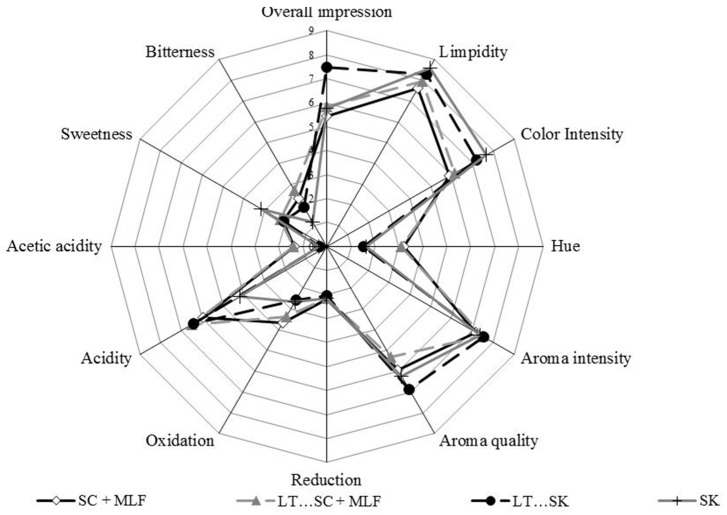
Results of the sensory analysis of bottled wines from different fermentation processes of *Saccharomyces cerevisiae* 88 alone (SC), sequential fermentation with *Saccharomyces cerevisiae* 88 and *L. thermotolerans* CONCERTO™ (LT…SC), sequential fermentation with *S. pombe*V2 and *L. thermotolerans* CONCERTO™ (LT…SK), *S. pombe*V2 alone (SK), and fermentations after malolactic fermentation with *Oenococcus oeni* 217 (+ MLF).

Figure 3 shows the spider web diagram of the average scores of the taste and olfactory attributes that were assessed. Large differences in the perception of acidity were recorded; this result agrees with the acidity parameters reported in Table 1, where combined fermentation between *L. thermotolerans* and *S. cerevisiae* after malolactic fermentation obtained the lowest pH and the highest concentration in lactic acid. Even though all wines contained residual sugar levels below 2 g/L, fermentation performed by *S. pombe* alone was perceived as sweeter than the others. This perception could be explained due to the increase of that sensory sensation, because of the new balance between acidity and sweetness after wine microbiological deacidification by *S. pombe*, without acidification by *L. thermotolerans* [63]. Alcoholic fermentation followed by malolactic fermentation produced a slightly stronger sensation of acetic acidity. This can be explained by the reported increase of acetic acid after malolactic fermentation (Table 1. Nevertheless, no serious faults were reported for any of the wines. The combination between *S. pombe* and *L. thermotolerans* received the best score in terms of overall impression from all tasters. Although all fermentations involving *S. pombe* achieved the main goals related to microbiological malic acid stabilisation. Other authors have reported fermentations by *L. thermotolerans* to possess higher concentrations of fruity esters and lower concentrations of higher alcohols than *S. cerevisiae* [2,43]. It also has been reported a loss of fruity character after lactic bacteria action [2]. Finally the combined fermentation of *S. pombe* and *L. Thermotolerans* showed a higher acidity due to the high lactic acid level (Table 1) which positively influenced the overall impression. Differences in the color intensity could be explained by the different anthocyanin profiles (Table 3).

## 3. Experimental Section

### 3.1. Microorganisms

The following yeast strains were used for the experimental fermentations: *Kluyveromyces thermotolerans* Concerto™ (Hansen, Hørsholm, Denmark; www.chr-hansen.com) belongs to the yeast species *L. thermotolerans*, *Saccharomyces cerevisiae* 88 (Spanish Type Culture Collection, Valencia, Spain) and *Schizosaccharomyces pombe* V2 (Chemistry and Food Technology department, Polytechnic University of Madrid, Madrid, Spain [35]. The strain of lactic acid bacteria used was *Oenococcus oeni* 217 (Spanish Type Culture Collection, Valencia, Spain).

### 3.2. Vinification

All fermentations used a must of *Vitis vinifera* L. cultivar Tempranillo grapes grown at the Entrena vineyard (Rioja Baja, Spain). The must was pasteurized at 105 °C for 5 min. A microvinification method similar to that described in the scientific literature was used [42]. Pasteurized must (4 L) was placed in a 5 L glass carboy, allowing adequate space for the release of carbon dioxide during fermentation. No sulfur dioxide was added. The sugar concentration was 226 g/L, pH = 3.64, primary amino nitrogen (PAN) 241 mg/L, malic acid 2.21 g/L, citric acid 0.24 g/L, lactic and acetic acid bellow 0.1 g/L. To provide nutrition 40 g/hL of Actimax Natura (Agrovín S.A., Ciudad Real, Spain) were added. Four treatments were used (all in triplicate): (i) inoculation of the must with *S. cerevisiae* 88 (10^7^ CFU/mL) alone (SC); (ii) inoculation of the must with *L. thermotolerans* Concerto™ (10^7^ CFU/mL) followed by *S. cerevisiae* 88 (10^7^ CFU/mL) 96 h later (LT…SC); (iii) inoculation of the must with *L. thermotolerans* Concerto™ (10^7^ CFU/mL) followed by *S. pombe* V2 (10^7^ CFU/mL) 96 h later (LT…SK); and (iv) inoculation of the must with *S. pombe* V2 (10^7^ CFU/mL) alone (SK). Yeasts were inoculated using 400 mL of sterilized must containing 10^8^ CFU/mL (determined using a Thomas chamber). To reach this population, 100 μL of each yeast suspension were cultivated in 10 mL of YEPD at 25 °C for 24 h. This procedure was repeated three times successively before the final inoculation of 4 mL in the inocula. All inoculations were performed in 500-mL flasks sealed with a Müller valve filled with 98% H_2_SO_4_ (Panreac, Barcelona, Spain), which allowed the release of CO_2_ while avoiding microbial contamination [64]. The temperature was maintained at 25 °C for 72 h before inoculation. The inoculations were developed under anaerobic conditions. All fermentations were performed in triplicate. All fermentation processes were carried out at 25 °C. When the sugar content was below 2 g/L, the wines were racked and stabilized for 7 days at 4 °C, after which the final product was bottled. Then, a concentration of 50 mg/L of sulfur dioxide in potassium metabisulfite form was added. Sealed bottles were placed horizontally in a climate chamber at 4 °C until the sensory evaluation took place. The wines fermented with *Saccharomyces cerevisiae* alone (SC) were stabilized and racked following the same procedure when the malolactic fermentation by *Oenococcus oeni* 217 (10^7^ CFU/mL) was finished in 2.8 L vessels at 18 °C. These wines remained under the same storage conditions described above, for one month before the tasting sessions took place.

### 3.3. Measurements of Biochemical Compounds and pH

Determination of glucose + fructose, l-malic acid, l-lactic acid, acetic acid, pyruvic acid, citric acid, acetaldehyde, urea, and glycerol concentrations (Table 1) were conducted using a Y15 Autoanalyser (Biosystems, Barcelona, Spain). The kits used to perform the analyses were obtained from Biosystems (www.biosystems.es). The alcohol content was determined using the boiling method GAB Microebu (http://shop.gabsystem.com). The pH was measured with a Crison pH Meter Basic 20 (Crison, Barcelona, Spain).

### 3.4. Microvinification Growth Kinetics

Aliquots were periodically taken aseptically during fermentation and further ten-fold serial dilutions were made. The yeast growth kinetics were monitored by plating 100 μL of the appropriate dilution on lysine media (non-*Saccharomyces* counts; [65]), YEPD media (total yeast counts; [66]) and YEPDActBzCl media (*Schizosaccharomyces* counts; [36]) with actidione and benzoic acid as the main inhibitors. In LT…SC fermentations, the population of *L. thermotolerans* was estimated by the difference between the YEPD and the Lysine media counts. In LT…SK fermentations, the population of *L. thermotolerans* was estimated by the difference between the YEPD and YEPDActBzCl media counts. Colonies were counted after growth at 30 °C for 48–72 h. Lactic acid bacteria was monitored in MRS agar (Oxoid, Basingstoke, UK).

### 3.5. Analytical Determination of Anthocyanins

Selected anthocyanins (Table 3) were analysed at the end of alcoholic and malolactic fermentations by high performance liquid chromatography using an Agilent Technologies series 1200 infinity series with a diode array detector (Hewlett-Packard, Palo Alto, CA, USA). Gradient of solvent A (water/formic acid, 95:5, *v/v*) and B (methanol/formic acid, 95:5, *v/v*) were used in a reverse-phase Poroshell 120 (Hewlett-Packard, Palo Alto, CA, USA) (5 cm; particle size 2.7 µm) as follows: 85% A and 15% B linear (1 mL/min) from 0 to 2 min, 85–50% A and 15–20% B linear (1 mL/min) from 2 to 10 min, 50% C and 50% B linear (1 mL/min) from 10–12 min and re-equilibration of the column from 12 to 13 min. Detection was performed by scanning in the 250–600 nm range. Quantification was performed by comparison against an external standard at 525 nm and expressed as a function of the concentration of malvidin-3-*O*-glucoside. The calibration was performed using malvidin-3-*O*-glucoside as an external standard, *R*^2^ > 0.999 (Extrasynthese, Geney, France). The sensitivity was higher than 0.1 mg/L. Controls of malvidin-3-*O*-glucoside were used to verify calibrations in each sequence. (Extrasynthese, Geney, France). Filtered wine samples of 20 μL (0.45 μm membrane filters made of cellulose methylic esters) (Teckorama, Barcelona, Spain) were injected in the HPLC apparatus. The different anthocyanins were identified by their retention times with respect to the majority anthocyanin malvidin-3-*O*-glucoside and by comparing the UV-Visible spectra with the data in the literature [25,44]. The following anthocyanins and pyronoanthocyanins were determined: delphinidin-3-*O*-glucoside (D3G). cyanidin-3-*O*-glucoside (C3G), petunidin-3-*O*-glucoside (Pt3G), peonidin-3-*O*-glucoside (Pn3G), malvidin-3-*O*-glucoside (M3G), Vitisine B (Vit B), Vitisine A (VitA), cyanidin-3-*O*-(6′′-acetylglucoside) (C3GAc), petunidin-3-*O*-(6′′-acetylglucoside) (Pt3GAc), peonidin-3-*O*-(6′′-acetylglucoside) (Pn3GAc), malvidin-3-*O*-(6′′-acetylglucoside) (M3GAc), cyanidin-3-*O*-(6′′-*p*-coumaroylglucoside) (C3GCm), petunidin-3-*O*-(6′′-p-coumaroylglucoside) (Pt3GCm), peonidin-3-*O*-(6′′-*p*-coumaroylglucoside) (Pn3GCm), malvidin-3-*O*-(6′′-*p*-coumaroylglucoside) (M3GCm), malvidin-3-*O*-glucoside-4-vinylphenol (M3G4Vp), malvidin-3-*O*-glucoside-4-vinylguaiacol (M3G4Vg), and malvidin-3-O-(6′′-*p*-coumaroylglucoside)-vinylphenol (M3GCm4Vp).

### 3.6. Color Measurements

An Y350 diode array spectophotometer (Biosystems, Barcelona, Spain) was used for the analysis. Samples were analyzed in a 1mm path length quartz cuvette and a range of 200–1100 nm. Absorbance at 420 nm, 520 nm, 620 nm was measured. Color intensity was calculated as the sum of absorbance at the three wavelengths, while tonality (hue) was calculated as the ratio between the absorbance at 420 nm and 520 nm.

### 3.7. Sensory Analysis

The final wines were assessed in a blind test by a panel of 15 experienced wine tasters, all of whom were staff members of the Chemistry and Food Technology Department (Madrid, Spain) and the Estación Enológica de Haro (Haro, Spain). Following the generation of a consistent terminology by consensus, three visual descriptors, four aromas and four taste attributes were chosen to describe the wines. The panellists used a 10 cm unstructured scale, from 0 (no perceived) to 10 (very strongly perceived) to rate the intensity of the 12 attributes.

### 3.8. Statistical Analyses

All statistical analyses were performed using PC Statgraphics v. 5 software (Graphics Software Systems, Rockville, MD, USA). The significance was set to *p* < 0.05 for the ANOVA matrix *F* value. A multiple range test was used to compare the means.

## 4. Conclusions

A combination of the *S. pombe* and *L. thermotolerans* selected yeast strains is an alternative to the traditional malolactic fermentation which positively affects the anthocyanin content of wine. The results from the fermentation trails showed positive differences in several parameters such as acetic acid, glycerol, acid profile, sensory evaluation, color and anthocyanin profile.

## Figures and Tables

**Table 1:** Final analysis of fermentations from original must of Tempranillo grapes (see Experimental section): *S. cerevisiae* 88 alone (SC), sequential fermentation with *S. cerevisiae* 88 and *L. thermotolerans* CONCERTO™ (LT…SC), sequential fermentation with *Schizosaccharomyces pombe* V2 and *L. thermotolerans* CONCERTO™ (LT…SK), *Schizosaccharomyces pombe* 4.5 alone (SK), and fermentations after a malolactic fermentation with *Oenococcus oeni* 217 (+ MLF).

Compounds	SC	SC + MLF	LT…SC	LT…SC + MLF	LT…SK	SK
l-Lactic Acid (g/L)	0.01 ± 0.01 a	1.21 ± 0.05 b	2.44 ± 0.14 c	3.62 ± 0.21 e	2.77 ± 0.19 d	0.01 ± 0.01 a
l-Malic Acid (g/L)	2.06 ± 0.03 b	0.01 ± 0.01 a	1.98 ± 0.06 b	0.01 ± 0.01 a	0.01 ± 0.01 a	0.01 ± 0.01 a
Acetic Acid (g/L)	0.31 ± 0.02 a	0.43 ± 0.04 b	0.29 ± 0.04 a	0.37 ± 0.06 ab	0.32 ± 0.04 a	0.34 ± 0.02 a
Glucose + Fructose (g/L)	1.11 ± 0.16 b	0.06 ± 0.02 a	1.33 ± 0.21 b	0.08 ± 0.04 a	1.23 ± 0.21 b	1.08 ± 0.14 b
Glycerol (g/L)	6.43 ± 0.02 a	6.48 ± 0.05 a	6.79 ± 0.05 b	6.82 ± 0.09 b	7.19 ± 0.06 c	7.55 ± 0.02 d
pH	3.68 ± 0.01 b	3.79 ± 0.03 c	3.53± 0.05 a	3.62 ± 0.07 a	3.50 ± 0.05 a	3.88 ± 0.02 d
Urea (mg/L)	2.28 ± 0.03 c	3.92 ± 0.06 e	2.06 ± 0.06 b	3.54 ± 0.19 d	0.08 ± 0.03 a	0.06 ± 0.02 a
Citric Acid (g/L)	0.22 ± 0.01 b	0.04 ± 0.02 a	0.21 ± 0.03 b	0.05 ± 0.04 a	0.22 ± 0.03 b	0.21 ± 0.01 b
Alcohol (% *v*/*v*)	13.27 ± 0.02 c	13.25 ± 0.04 c	13.04 ± 0.04 b	13.09 ± 0.07 b	12.83 ± 0.06 a	12.89 ± 0.03 a
Acetaldehyde (mg/L)	55.31 ± 1.82 c	2.18 ± 0.33 a	36.42 ± 2.98 b	1.99 ± 0.24 a	63.58 ± 3.78 d	76.42 ± 2.58 e
Pyruvic Acid (mg/L)	89.45 ± 5.43 c	27.52 ± 2.43 a	102.31 ± 7.82 d	36.73 ± 3.16 b	198.63 ± 8.56 e	286.77 ± 6.41 f

Results are the mean ± SD of three replicates. Means in the same row with the same letter are not significantly different (*p* < 0.05).

**Table 2:** Color measurements in the wines produced by the different fermentation assays: *S. cerevisiae* 88 alone (SC), sequential fermentation with *S. cerevisiae* 88 and *L. thermotolerans* CONCERTO™ (LT…SC), sequential fermentation with *S. pombe* V2 and *L. thermotolerans* CONCERTO™ (LT…SK), *S. pombe* V2 alone (SK), and fermentations after malolactic fermentation with *Oenococcus oeni* 217 (+ MLF).

Compounds	SC	SC + MLF	LT…SC	LT…SC + MLF	LT…SK	SK
420 nm	0.05 ± 0.01 a	0.06 ± 0.01 a	0.05 ± 0.01 a	0.06 ± 0.01 a	0.06 ± 0.01 a	0.06 ± 0.01 a
520 nm	0.12 ± 0.01 b	0.08 ± 0.01 a	0.11 ± 0.01 b	0.09 ± 0.01 a	0.12 ± 0.01 b,c	0.14 ± 0.01 c
620 nm	0.02 ± 0.01 a	0.01 ± 0.01 a	0.02 ± 0.01 a	0.01 ± 0.01 a	0.02 ± 0.01 a	0.02 ± 0.01 a
CI	0.19 ± 0.01 ab	0.15 ± 0.01 a	0.18 ± 0.01 ab	0.16 ± 0.01 a	0.20 ± 0.01 b,c	0.22 ± 0.01 c
Hue	0.41 ± 0.02 a	0.75 ± 0.02 b	0.45 ± 0.02 a	0.66 ± 0.02 b	0.50 ± 0.02 a	0.42 ± 0.02 a

Results are the mean ± SD of three replicates. Means in the same row with the same letter are not significantly different (*p* < 0.05).

**Table 3:** Final analysis of anthocyanins from fermentations by *S. cerevisiae* 88 alone (SC), sequential fermentation with *S. cerevisiae* 88 and *L. thermotolerans* CONCERTO™ (LT…SC), sequential fermentation with *S. pombe* V2 and *L. thermotolerans* CONCERTO™ (LT…SK), *Schizosaccharomyces pombe* V2 alone (SK), and fermentations after malolactic fermentation with *Oenococcus oeni* 217 (+ MLF).

Compounds (mg/L)	SC	SC + MLF	LT…SC	LT…SC + MLF	LT…SK	SK
D3G	14.63 ± 0.56 b	11.70 ± 0.56 a	19.32 ± 1.06 d	15.42 ± 1.28 b,c	17.43 ± 1.22 c	15.73 ± 0.61 b
C3G	0.25 ± 0.01 a	-	0.66 ± 0.09 d	0.29 ± 0.14 a,b	0.54 ± 0.18 c	0.36 ± 0.04 b
Pt3G	19.06 ± 0.38 c	14.29 ± 0.53 a	21.48 ± 0.62 d	16.75 ± 0.81 b	21.77 ± 0.91 d	19.32 ± 0.44 c
Pn3G	6.44 ± 0.18 b	4.83 ± 0.29 a	12.06 ± 1.46 e	8.89 ± 1.66 c,d	9.86 ± 0.73 d	7.82 ± 0.33 c
M3G	101.16 ± 2.88 c	70.91 ± 3.47 a	106.31 ± 3.55 c	79.42 ± 4.12 b	102.46 ± 3.86 c	98.44 ± 3.14 c
VitA + VitB	4.89 ± 0.34 a		5.11 ± 0.46 a		5.98 ± 0.63 b	6.93 ± 0.41 c
VitA	4.35 ± 0.30 b	3.02 ± 0.36 a	4.56 ± 0.31 b	3.41 ± 0.39 a	5.27 ± 0.53 c	5.98 ± 0.34 d
VitB	0.54 ± 0.03 c	0.16 ± 0.06 a	0.42 ± 0.05 b	0.11 ± 0.08 a	0.95 ± 0.10 d	1.11 ± 0.06 e
VitA-Ac	0.66 ± 0.05 c	0.41 ± 0.09 a	0.52 ± 0.07 b	0.38 ± 0.11 a	0.86 ± 0.12 d	1.22 ± 0.09 e
C3GAc	1.10 ± 0.07 d	0.90 ± 0.11 c	0.57 ± 0.04 b	0.44 ± 0.07 a	1.21 ± 0.08 d,e	1.32 ± 0.05 e
Pt3GAc	4.70 ± 0.29 bc	3.76 ± 0.45 ab	4.41 ± 0.21 bc	3.51 ± 0.32 a	4.93 ± 0.29 c	5.02 ± 0.23 c
Pn3GAc	3.01 ± 0.06 c	1.68 ± 0.08 a	3.33 ± 0.16 d	2.16 ± 0.24 b	3.29 ± 0.20 c,d	3.21 ± 0.08 d
M3GAc	23.37 ± 0.77 c	15.55 ± 1.06 a	25.62 ± 1.02 d	19.26 ± 1.27 b	25.23 ± 1.21 c,d	24.39 ± 0.82 c,d
C3GCm	0.62 ± 0.03 b	0.39 ± 0.06 a	0.71 ± 0.05 c	0.49 ± 0.09 a	0.69 ± 0.08 b,c	0.61 ± 0.04 b
Pt3GCm	1.68 ± 0.04 b	1.14 ± 0.09 a	2.33 ± 0.07 d	1.77 ± 0.12 b	2.06 ± 0.09 c	1.78 ± 0.03 b
Pn3GCm	0.18 ± 0.01 a	-	0.26 ± 0.03 b	-	0.29 ± 0.06 b,c	0.33 ± 0.03 c
M3GCm	13.44 ± 0.19 b	9.18 ± 0.28 a	17.43 ± 0.42 d	14.26 ± 0.86 b,c	15.55 ± 0.54 c	13.51 ± 0.22 b
M3G4Vp	0.00 ± 0.00 a	0.00 ± 0.00 a	0.00 ± 0.00 a	0.00 ± 0.00 a	0.00 ± 0.00 a	0.00 ± 0.00 a
M3G4Vg	0.00 ± 0.00 a	0.00 ± 0.00 a	0.00 ± 0.00 a	0.00 ± 0.00 a	0.00 ± 0.00 a	0.00 ± 0.00 a
M3GAc4Vp	0.00 ± 0.00 a	0.00 ± 0.00 a	0.00 ± 0.00 a	0.00 ± 0.00 a	0.00 ± 0.00 a	0.00 ± 0.00 a
M3GCm4Vp	0.00 ± 0.00 a	0.00 ± 0.00 a	0.00 ± 0.00 a	0.00 ± 0.00 a	0.00 ± 0.00 a	0.00 ± 0.00 a
Total anthocyanins	200.08	137.92	225.1	166.56	218.37	207.08

Results represent the mean ± SD for three replicates. Means in the same row with the same letter are not significantly different (*p* < 0.05).
